# Use of glucagon in severe hypoglycemia is scarce in most countries, and has not been expanded by new ready-to-use glucagons

**DOI:** 10.1186/s13098-022-00950-6

**Published:** 2022-12-23

**Authors:** Antonio E. Pontiroli, Manfredi Rizzo, Elena Tagliabue

**Affiliations:** 1 Dipartimento Di Scienze Della Salute, Università Degli Studi Di Milano, Milan, Italy; 2 Promise Department, School of Medicine, University of Palermo, Palermo, Italy; 3 Value-Based Healthcare Unit, IRCCS MultiMedica, Milan, Italy

**Keywords:** Hypoglycemia, Severe hypoglycemia, Glucagon, Nasal glucagon, Emergency kits, Dasiglucagon, Non-acqueous glucagon solutions, Persons with type 1 diabetes, IDF diabetes atlas, IQVIA database, Sales of glucagon units

## Abstract

Glucagon (traditional kits for intramuscular administration, Glucagon and Glucagen), although recommended as a remedy for severe hypoglycemia (SH), has been reported to be under-utilized, likely because of technical problems. The aims of this study were to evaluate the use of glucagon in persons with type 1 diabetes in several countries, and to investigate if the availability of new ready-to-use glucagons (Baqsimi, Gvoke, Zegalogue, years 2019 to 2021) has expanded the overall use of glucagon. The source of data was IQVIA-MIDAS (units of glucagon sold), while data on persons with type 1 diabetes in countries were derived from IDF Diabetes Atlas. The use of glucagon has been steady from 2014 to 2019, with a small but significant increase from 2019 to 2021, paradoxically only in countries where new ready-to-use glucagons were not available. The use of glucagon has always been ten fold greater in countries where new ready-to-use glucagons became available than in the other countries (population 108,000,000 vs 28,100,000, 480,291 vs 182,018 persons with type 1 diabetes). A significant correlation was observed in all years between units of glucagon and persons with type 1 diabetes. Availability of new ready-to-use glucagons was associated with a small increase of sales, due only to new ready-to-use glucagons themselves. The use of glucagon (any type) remains low, approximately 1/10 of persons with type 1 diabetes. We conclude that use of glucagon is scarce in most countries, and so far has not been expanded by new ready-to-use glucagons such as the ones considered in this study.

**Supplementary Information:**

The online version contains supplementary material available at 10.1186/s13098-022-00950-6.

## Introduction

Hypoglycemia, symptomatic hypoglycemia, and severe hypoglycemia (SH, a circumstance where the patient can be unconscious, and requires the assistance of someone else), are frequent in persons with type 1 (T1D) and type 2 diabetes (T2D) who use insulin, or in T2D patients on sulphonylureas; frequency of hypoglycemia is greater in T1D than in T2D patients, and depends on regimens of insulin administration, on age and associated medical problems [1–3]. The experience of hypoglycemia leads to fear of hypoglycemia, that in turn can limit optimal glycemic control in many children and adolescents as well as in adults with type 1 diabetes [4]. More importantly, hypoglycemia can lead to cardiovascular accidents [5], to falls and trauma [6], to cognitive impairment [7].

Hypoglycemia requires immediate correction, and can be managed, depending on its severity, through ingestion of glucose, glucagon injection, or intravenous glucose solution [8]. Glucagon is recommended as out of hospital remedy for SH, when glucose by the oral route might be dangerous. It can be administered by intramuscular (IM), intravenous, or subcutaneous injection, is safe and effective, and has few and predictable side effects [8]; emergency glucagon kits are available in several countries since the early 2000s or even before. However, glucagon has to be reconstituted immediately prior to administration because glucagon solutions are not stable [9]. Therefore, correct administration of glucagon requires reconstitution of a solution, and this, especially under stress or emergency situations, is difficult to obtain for untrained patients or care-givers [9].

This is one of the reasons why, compared with the expectations, glucagon injection is under-utilized in some countries [10–12]. In 1997, a survey showed that patients would have been enthusiastic on the idea that easily administered formulations would substantially expand the use of glucagon to prevent or to treat hypos, but a survey showed that 75% adult patients with diabetes did not carry a glucagon emergency kit [9]. Therefore, the unmet need was a easy-to-use glucagon emergency kit for trained and untrained patients and care-givers. In 1983 it was shown that intranasal glucagon raises blood glucose, and in 1989 it was shown that nasal glucagon is as effective as IM glucagon in the treatment of spontaneous hypoglycemia in adults; later it was shown that intranasal glucagon is effective in insulin-induced hypoglycemia in children and in adults [9]. The project was developed in the years 2010–2015, and led to approval of nasal glucagon (Baqsimi) in US, Canada, Europe, Japan in 2019–2020. Other approaches to overcome instability of glucagon solutions were developed, such as stable non-acqueous glucagon solutions (Gvoke, 2020) [13] and modified glucagon molecules (Zegalogue, 2021) [14]. The aim of this study is to yield a picture of use of glucagon in several countries, including both traditional emergency kits and new ready-to-use glucagon formulations, and to understand if the use of the new ready-to-use glucagons (Baqsimi, Gvoke, and Zegalogue) has expanded the use of glucagon as remedy for hypoglycemia.

## Materials and methods

We estimated global glucagon sales in the years2014-2021 using the IQVIA-Multinational Integrated Data Analysis System (IQVIA-MIDAS®) database [15]. IQVIA-MIDAS is a commercial database containing data from pharmacy retail sales throughout the supply chains, including overall glucagon volume sold to retailers and hospital pharmacies by wholesalers. The proportion of wholesalers contributing data to IQVIA-MIDAS varies between represented countries and IQVIA adjusts the reported data based on the market share of participating wholesalers, to provide estimates of total sales in the sectors represented in each country. This dataset contains annual pharmaceutical sales data for 75 countries/regions. Annual sales of glucagon (including information on component glucagon, formulation, trade name and manufacturer) are recorded for each country. Glucagon sales were expressed in standard units (SU), with 1 SU defined by IQVIA as one emergency kit, nasal set, or injectable easy-to-use glucagon. The IQVIA-MIDAS® analytics platform is validated annually by calculation of a ‘global precision index’ (94.3% in 2017) [15]. Data of distribution and sales of glucagon, [old formulations (Glucagon, Glucagen) and new ready-to-use glucagons (Baqsimi, Gvoke, Zegalogue)] were requested to IQVIA for Algeria, Argentina, Australia, Austria, Bangladesh, Belarus, Belgium, Bosnia, Brazil, Bulgaria, Central America, Canada, Chile, China, Colombia, Croatia, Czech Republic, Dominican Republic, Ecuador, Egypt, Estonia, Finland, French West Africa, France, Germany, Greece, Hong Kong, Hungary, India, Indonesia, Ireland, Italy, Japan, Jordan, Kazakhstan, Korea, Kuwait, Latvia, Lebanon, Lithuania, Luxembourg, Malaysia, Mexico, Morocco, Netherlands, New Zealand, Norway, Pakistan, Peru, Philippines, Poland, Portugal, Puerto Rico, Romania, Russian Federation, South Africa, Saudi Arabia, Serbia, Singapore, Slovakia, Slovenia, Spain, Sri Lanka, Sweden, Switzerland, Taiwan, Thailand, Tunisia, Turkey, UAE, UK, Uruguay, US, Venezuela, Vietnam. Data for European countries where Baqsimi was eventually available (Belgium, Bulgaria, Slovakia, France, Germany, Greece, Hungary, Italy, Luxembourg, Netherlands, Norway, Poland, Portugal, Romania, Spain) were grouped under the label Europe, at difference from UK, Turkey, and Ireland, where Baqsimi did not become available.

New ready-to-use glucagons were available in four specified regions (US, Canada, Europe, and Japan, Group A), their data were analyzed by country and together, as well as data for countries where the new formulations were not available (Group B). Data collected referred only to retail sales; since the use of glucagon in hospital settings is not only limited to management of hypoglycemia, these data were deliberately ignored. Data were always expressed as referred to persons with type 1 diabetes.

The period analyzed included 2014–2021 to compare sales before and after availability of Baqsimi, Gvoke, Zegalogue. This was intended to understand the spontaneous pattern of traditional glucagon kits for a long enough period. Data on number of persons with type 1 diabetes in different countries were derived from the 2021 edition of the IDF diabetes atlas [16]; however, these data should be interpreted with caution, since methods of calculation varied from year to year (prevalence × 1000 in 2013, × 100.000 for 2015 and 2017, absolute numbers for 2019 and 2021) [16]. A very recent publication reported significantly higher estimates for the prevalence of type 1 diabetes in different countries in 2021 [17]. This study was considered for completeness, and correlation between sales and persons with type 1 diabetes is presented in the Additional file 1. This study followed the guidelines for cohort studies, described in the Strengthening the Reporting of Observational Studies in Epidemiology (STROBE) reporting guideline [18].

## Ethics approval and informed consent

IQVIA-MIDAS data are not ad hoc collected for studies purposes and there is no sponsor. Being so, all the analyses conducted on IQVIA-MIDAS data do not require an approval by an Ethical Committee.

## Statistical analysis

No statistical sample size calculation was performed a priori, and sample size was equal to the number of glucagon sales in units during the study period. All sales of different trade marks for each country/region were grouped to yield a single number for each country/region. Continuous variables are presented as totals plus mean and standard deviation. Data of different countries/regions were grouped according to the eventual registration of new glucagon drugs. Non-parametric Mann–Whitney test was used to compare glucagon sales between two groups, ie with/without availability of new glucagon drugs. Changes within groups were analyzed by the non-parametric Wilcoxon test. Sales per person were also calculated, and pairwise correlations between items of interest were calculated. All statistical tests were 2-tailed, and statistical significance was defined as p < 0.05. All statistical analyses were performed employing Stata 12 for Macintosh (Stata Corporation, College Station, Texas).

## Results

Sales in units of any type of glucagon (old and new drugs) in the period 2014–2021 in the four considered regions (where new drugs were available, Group A) and in the other countries (Group B) are shown in Table 1. Global sales were always around ten fold higher in Group A than in Group B (Table 1A). Persons with diabetes per one unit of glucagon were not different between the two Groups (Table 1B). Similarly, units of glucagon per person with diabetes were not different between the two Groups (Table 1C). Data from China were not considered because of the unclear behaviour through different years.

**Table 1:** Sales of glucagon (any formulation, millions of units), persons with type 1 diabetes per one unit of glucagon, and Units of glucagon per person with type 1 diabetes during the period 2014–2021. Totals, mean ± SD

A) sales of glucagon units									
Year	2014	2015	2016	2017	2018	2019	2020	2021	Persons with type 1 diabetes
New drugs available (Group A)^a^									
Total	2.054	2.129	2.214	2.184	2.177	2.425	2.419	2.731	480,291
Mean	0.513°	0.532°	0.553°	0.546°	0.544°	0.606°	0.605°	0.683°	120,072
SD	0.373	0.394	0.436	0.437	0.439	0.526	0.498	0.559	136,117
New drugs not available (Group B)^b^									
Total	0.292	0.307	0.339	0.333	0.349	0.372	0.348	0.395	182,018
Mean	0.036 # §	0.032 # §	0.042 §	0.042 §	0.043 §	0.046	0.043	0.049	20,224
SD	0.038	0.042	0.046	0.045	0.045	0.049	0.045	0.040	18,465
Grand total									
Total	2.346	2.436	2.552	2.518	2.526	2.797	2.767	3.126	662,309
Mean	0.195 # §	0.203 # §	0.212 §	0.209 §	0.210 §	0.233	0.231	0.260	50,496
SD	0.307	0.320	0.341	0.339	0.339	0.391	0.381	0.429	84,616

B) persons with type 1 diabetes per one unit of glucagon									
New drugs available (Group A)^a^									
Mean	20.579	20.774	20.207	18.628	17.869	15.792	14.534	17.633	
SD	25.733	30.826	29.173	27.053	25.432	20.766	17.479	25.589	
New drugs not available (Group B)^b^									
Mean	9.202°	5.344	5.911	5.014	5.422	4.429	4.221	5.356	
SD	7.952	4.033	4.419	2.990	3.303	3.054	2.942	4.474	
Grand Total									
Mean	13.339°	10.487	10.677	9.552	9.572	8.217	7.659	9.448	
SD	16.418	18.089	17.149	15.818	14.863	12.443	10.706	15.095	

C) Units of glucagon per one person with type 1 diabetes									
New drugs available (Group A)^a^									
Mean	0.153	0.204	0.199	0.202	0.201	0.174	0.152	0.165	
SD	0.172	0.221	0.218	0.203	0.202	0.147	0.113	0.106	
New drugs not available (Group B)^b^									
Mean	0.552	1.088	1.152	0.735	0.629	0.839	0.979	0.677	
SD	0.803	2.010	2.244	1.129	0.905	1.282	1.543	0.915	
Grand total									
Mean	0.407	0.793	0.834	0.558	0.487	0.618	0.703	0.507	
SD	0.661	1.665	1.854	0.944	0.759	1.077	1.298	0.774	

^a^ Group A: Canada, US, Europe, Japan;

^b^Group B: Argentina, Australia, China, Ireland, New Zealand, Russia, South Africa, Turkey, UK

p < 0.05 Group A vs Group B; # p < 0.05 vs year 2020 and year 2021; § p < 0.05 vs year 2019

**Fig. 1:**
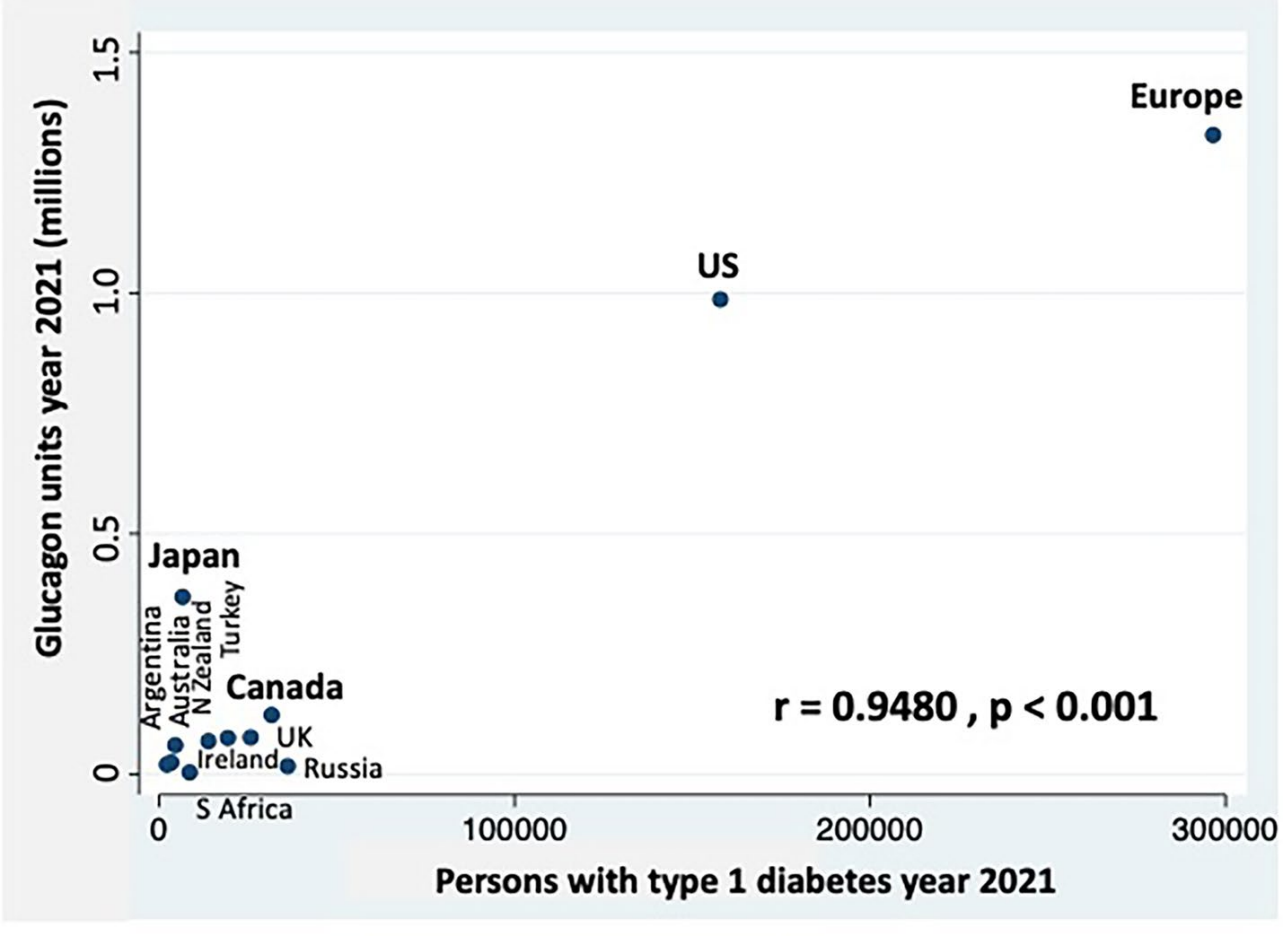
Sales of glucagon (any kind, millions of units) and persons with type 1 diabetes in the year 2021

A small but significant increase of sales took place in the period 2019–2021, in total and in Group B; similarly, persons per one unit of glucagon decreased, in total and in Group B (Table 1B). Figure 1 shows a significant correlation between persons with type 1 diabetes and units of glucagon sold in 2021, independently of the approval of new ready-to-use glucagons.

The Additional file 1contains details of persons with diabetes per country in various years (Additional file 1: Table S1), of persons with type 1 diabetes per country in various years (Additional file 1: Table S2), of correlations between persons with type 1 diabetes and units of glucagon sold in years 2014–2019 (Additional file 1: Figure S1). During the period 2019–2021 sales showed a small increase in general, and in Group B. In Group A, sales did not change if the three new ready-to-use glucagons were excluded from calculations, or showed a decrease (Additional file 1: Table S3). This is interpreted as a small increase of sales only due to the new ready-to-use glucagons, at the expense of older formulations.

Data from different countries/regions showed that units sold per person were 0.507 ± 0.774 in 2021, with no differences between countries of Group B (0.677 ± 0.915) or of Group A (0.165 ± 0.106). These figures are not significantly different from previous years, indicating that the use of glucagon has not signifcantly changed during the period 2014–2021; this is likely due to the increase in prevalence of type 1 diabetes and to a small parallel increase of sale of glucagons. If new data on the prevalence of type 1 diabetes in 2021 [17] are confirmed, the units sold per person would be aven lower than reported in this paper (Additional file 1: Table S2 and Additional file 1: Figure S2).

## Discussion

To our knowledge, this is the first analysis of use of glucagon for SH in several countries/ regions around the world. Use of traditional glucagon kits is low, as reported in previous studies [10–12], and the new finding is that the use is low in the totality of countries examined, so that a significant correlation was found between persons with diabetes or type 1 diabetes and units of glucagon sold each year. Units sold per person from 2014 to 2021 were 0.407 ± 0.661 to 0.507 ± 0.774 (Mean ± SD) in 2021, that means that < 50% of persons with diabetes had glucagon available, and probably even fewer persons were treated with glucagon for SH.

We know that old formulations remain unpopular because of problems connected with the preparation and administration of glucagon [9–12], and the general hope was that the availability of new ready-to-use glucagon drugs might expand its use. Units sold were always approximately tenfold greater in the countries/regions where new ready-to-use glucagon drugs (Baqsimi, Gvoke, Zelagogue) were available in 2019–2021 than in remaining countries/regions; however, this did not mean a more frequent use of glucagon, as reflected by the significant correlation between persons with type 1 diabetes and units of glucagon sold, independently from approval of new ready-to-use drugs. Also, the availability of new ready-to-use drugs led to a small increase of sales of glucagon units, only due to new drugs. As a matter of fact, sales without the new ready-to-use glucagons were stable or decreased, and the increase of use was paradoxically significant only in countries/regions where the new drugs were not available, supporting a natural trend to a greater use of glucagon, not driven by availability of new glucagon drugs.

## Limitations

The first limitation is that data of China could not be considered because of the unclear behaviour through different years. Also, the availability of new ready-to-use drugs only dates 2019 for Baqsimi, to 2020 for Gvoke, and to 2021 for Zegalogue. For this reason these data represent only an early evaluation, to be confirmed during the next years. Also, all ready-to-use glucagons were launched during the Covid-19 pandemic; the pandemic has had a very negative impact on the ability of pharmaceutical companies to meet and educate physicians and diabetes educators. It probably also had an impact on frequency of patient visits with care providers (i.e., reduced prescribing) and on the purchasing behavior of patients (i.e., not traveling therefore less perceived need for an emergency glucagon). Finally, one should consider that the ready-to-use glugagons are very recent developments, and that most pharmaceutical companies initially target the highest-value markets—the US, Europe, Japan followed by Canada, Australia and other countries. The fact that the products are not in other countries is probably not related to approval from local authorities, but to the fact that the sponsor has not yet submitted to local authorities of those countries. The final limitation is that data on real prevalence of type 1 diabetes are still limited, being probably tenfold higher than usually reported, according to recent modelling based on incidence and mortality data [17]. If these data are confirmed, this would mean that figures of distribution and use of glucagon are even lower than calculated in this paper.

## Conclusion

The use of glucagon is low in all countries/regions examined, and so far the advent of new ready-to-use glucagons has slightly increased sales of glucagon, but only of the new drugs themselves, not of glucagon in general. It is anticipated that problems linked to traditional glucagon emergency kits will only be challenged by new ready-to-use glucagons such as the ones considered here, or by future new ready-to-use glucagon molecules.

## Supplementary Information

**Additional file 1: Table S1.** Persons with diabetes per country in various years. **Table S2.** Persons with type 1 diabetes per country in various years. Absolute prevalence. **Table S3.** Glucagon sales (any type, millions of units) in 2019-2021 in countries where new ready-to-use glucagons were or were not available in 2019-2021. Only retail data are considered. Totals and mean ± SD are reported. On the left: sales in countries where new drugs were (Group A) or were not (Group B) available in 2019-2021. On the right: sales in the same countries when new drugs are not considered. **Table S4.** Glucagon units (any type) by country. Totals and mean ± SD. Retail and hospital sales are considered together. **Table S5.** Commercial sales of glucagon formulations (any type) by country. Retail and hospital sales are considered together. **Figure S1.** Persons with type 1 diabetes and glucagon sales (any type, millions of units) in years 2014-2019.**Figure S2.** Persons with type 1 diabetes and glucagon sales (any type, millions of units) in year 2021; comparison between data on prevalence of type 1 diabetes according to IDF Atlas and to the recent paper (Lancet Diabetes Endocrinol 2022; 10:741).

## Data Availability

The data that support the findings of this analysis are available from IQVIA-MIDAS, but restrictions apply to the availability of these data, which were used under license for the current analysis, and so are not publicly available. Data are, however, available from the corresponding author upon reasonable request and with permission of IQVIA-MIDAS.
