# Supplementary material for: Use of glucagon in severe hypoglycemia is scarce in most countries, and has not been expanded by new ready-to-use glucagons
Source: Diabetol Metab Syndr. 2022 Dec 23;14:193. doi: 10.1186/s13098-022-00950-6 (PMC9780089; doi:10.1186/s13098-022-00950-6)
Supplement: Supplementary file 1 — Additional file 1: Table S1. Persons with diabetes per country in various years. Table S2. Persons with type 1 diabetes per country in various years. Absolute prevalence. Table S3. Glucagon sales (any type, millions of units) in 2019-2021 in countries where new ready-to-use glucagons were or were not available in 2019-2021. Only retail data are considered. Totals and mean ± SD are reported. On the left: sales in countries where new drugs were (Group A) or were not (Group B) available in 2019-2021. On the right: sales in the same countries when new drugs are not considered. Table S4. Glucagon units (any type) by country. Totals and mean ± SD. Retail and hospital sales are considered together. Table S5. Commercial sales of glucagon formulations (any type) by country. Retail and hospital sales are considered together. Figure S1. Persons with type 1 diabetes and glucagon sales (any type, millions of units) in years 2014-2019.Figure S2. Persons with type 1 diabetes and glucagon sales (any type, millions of units) in year 2021; comparison between data on prevalence of type 1 diabetes according to IDF Atlas and to the recent paper (Lancet Diabetes Endocrinol 2022; 10:741). [file 13098_2022_950_MOESM1_ESM.docx]

**Use of glucagon in severe hypoglycemia is scarce in most countries, and has not been expanded by new ready-to-use glucagons.**

Antonio E. Pontiroli, Manfredi Rizzo, Elena Tagliabue

Supplementary Appendix

Supplementary Table 1. Persons with diabetes per country in various years.

|  | Persons with diabetes per year | | | | |
| --- | --- | --- | --- | --- | --- |
| Country/Region | 2013 | 2015 | 2017 | 2019 | 2021 |
| Argentina | 1,600,000 | 1,700,000 | 1,800,000 | 1,800,000 | 1,800,000 |
| Australia | 1,600,000 | 1,100,000 | 1,100,000 | 1,300,000 | 1,490,000 |
| Canada | 2,400,000 | 2,500,000 | 2,600,000 | 2,800,000 | 3,000,000 |
| China | 98,000,000 | 110,000,000 | 110,000,000 | 120,000,000 | 141,000,000 |
| Europe | 56,000,000 | 60,000,000 | 58,000,000 | 59,000,000 | 61,800,000 |
| Ireland | 207,490 | 171,800 | 141,500 | 148,200 | 140,000 |
| Japan | 7,200,000 | 7,200,000 | 7,200,000 | 7,400,000 | 11,000,000 |
| N Zealand | 34,280 | 28,590 | 32,610 | 25,980 | 26,800 |
| Russia | 11,000,000 | 12,000,000 | 8,500,000 | 8,300,000 | 7,390,000 |
| S Africa | 2,600,000 | 2,300,000 | 1,800,000 | 4,600,000 | 4,230,000 |
| Turkey | 7,000,000 | 6,300,000 | 6,700,000 | 6,600,000 | 9,020,000 |
| UK | 3,000,000 | 2,900,000 | 2,700,000 | 2,700,000 | 3,990,000 |
| US | 24,000,000 | 29,000,000 | 30,000,000 | 31,000,000 | 32,200,000 |
| *Total* | *214,641,770** | *235,200,390* | *230,574,110** | *245,674,180* | *277,398,800* |

Absolute numbers, with no specification as to type 1 or type 2 diabetes; * p < 0.05 vs year 2021

**Supplementary Table 2.**

Persons with type 1 diabetes per country in various years. Absolute prevalence

|  | Persons with type 1 diabetes per year | | | | | New estimate # |
| --- | --- | --- | --- | --- | --- | --- |
| Country/Region | 2013 | 2015 | 2017 | 2019 | 2021 | 2021 |
| ARGENTINA | 8,482 | 7,929 | 8,800 | 8,619 | 8,581 | 82,015 |
| australia | 3,630 | 6,300 | 12,299 | 12,969 | 13,854 | 122,139 |
| CANADA | 16,591 | 23,110 | 24,000 | 21,573 | 19,331 | 276,284 |
| CHINA | 59,044 | 33,500 | 51,254 | 54,040 | 56,013 | 430,647 |
| Europe | 129,500 | 140,000 | 150,000 | 160,000 | 296,500 | 2,927,365 |
| IRELAND | 1,273 | 3797 | 3430 | 3250 | 3,364 | 25,640 |
| JAPAN | 3,001 | 7,202 | 7,234 | 8,483 | 6,586 | 77,874 |
| N ZEALAND | 848 | 2,859 | 2,000 | 2,528 | 2,172 | 17,600 |
| RUSSIA | 9,028 | 18,500 | 20,000 | 35,725 | 36,140 | 321,460 |
| S AFRICA | 3,307 | 2,280 | 1,500 | 1,599 | 4,516 | 30,075 |
| TURKEY | nd | 6399 | 11200 | 39130 | 25753 | 137,071 |
| UK | 11,805 | 19,800 | 19,900 | 25,953 | 31,625 | 403,608 |
| US | 42,194 | 84,100 | 100,000 | 120,000 | 157,874 | 1,414,441 |
| Total | 288,703 | 355,776 § | 411,617 * § | 493,869 * | 662,309 * | 5,836,003 |
| Mean | 24,058 | 27,367 | 31,662 | 37,989 | 50,946 | 448,923 |
| SD | 37,772 | 40,362 | 44,619 | 48,681 | 84,616 | 833,723 |

Source: IDF Diabetes Atlas: Global, regional and country-level diabetes prevalence estimates for 2021 and

projections for 2045, Diabetes Res Clin Pract, 2022; 183: 109119. The data must be interpreted with caution,

since methods of calculation varied from year to year (prevalence x 1000 in 2013, x 100.000 for 2015 and 2017,

absolute numbers for 2019 and 2021); * p < 0.05 vs year 2013; § p < 0.05 vs year 2021

# according to the paper under reference 18 (Lancet Diabetes Endocrinol. 2022; 10: 741-760).

**Supplementary Figure 1**.

Persons with type 1 diabetes and glucagon sales (any type, millions of units) in years 2014-2019


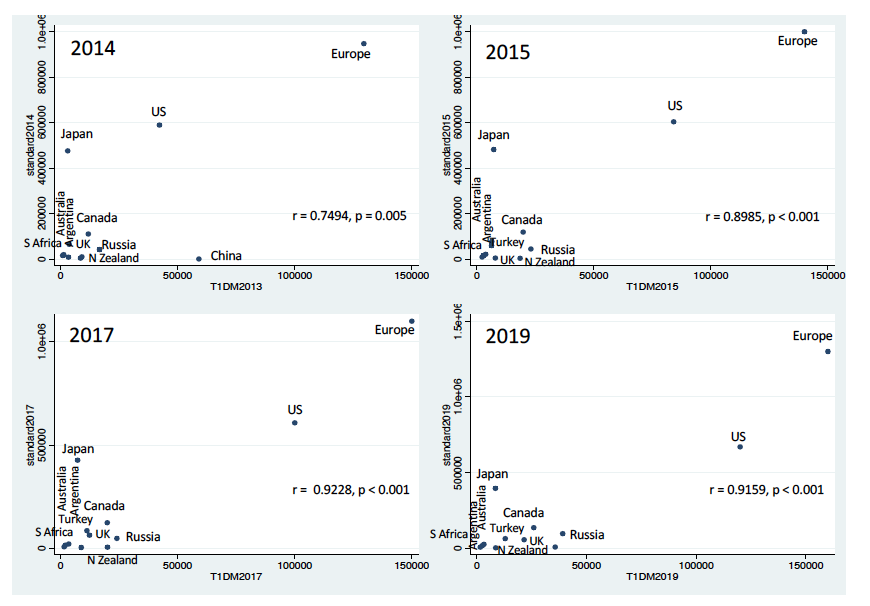


**Supplementary Figure 2.**

Persons with type 1 diabetes and glucagon sales (any type, millions of units) in year 2021; comparison between

data on prevalence of type 1 diabetes according to IDF Atlas and to the recent paper (Lancet Diabetes Endocrinol 2022;10:741)

**
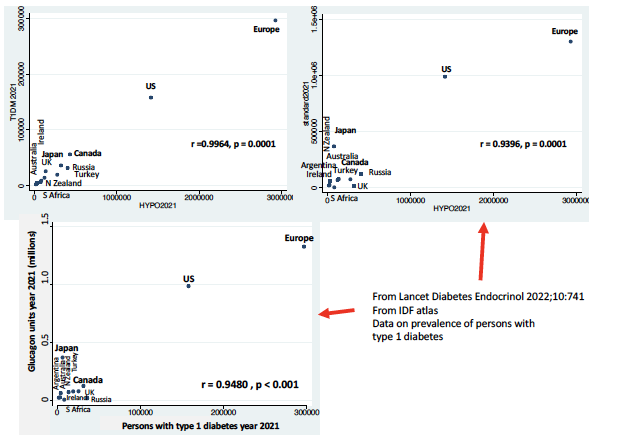
**

**Supplementary Table 3.**

Glucagon sales (any type, millions of units) in 2019-2021 in countries where new ready-to-use glucagons were or were not available in 2019-2021. Only retail data are considered. Totals and mean ± SD are reported.

On the left: sales in countries where new drugs were (Group A) or were not (Group B) available in 2019-2021.

On the right: sales in the same countries when new drugs are not considered.

|  | New ready-to-use glucagons are considered | | | New ready-to-use glucagons not considered | | |
| --- | --- | --- | --- | --- | --- | --- |
| Year | 2019 | 2020 | 2021 | 2019 | 2020 | 2021 |
| *New drugs available (Group A) ** | | | | | | |
| Total | 2.425 | 2.419 | 2.731 | 2.297 | 1.981 | 2.003 |
| Mean | 0.606 ° | 0.605 ° | 0.683 ° | 0.574° | 0.495° | 0.501° |
| SD | 0.526 | 0.498 | 0.559 | 0.513 | 0.463 | 0.484 |
| *New drugs not available (Group B) *** | | | | | | |
| Total | 0.372 | 0.348 | 0.395 | 0.372 | 0.348 | 0.395 |
| Mean | 0.046 | 0.043 | 0.049 | 0.046 | 0.043 | 0.049 |
| SD | 0.049 | 0.045 | 0.040 | 0.049 | 0.045 | 0.040 |

* Group A: Canada, US, Europe, Japan;

** Group B: Argentina, Australia, China, Ireland, New Zealand, Russia, South Africa, Turkey, UK

° p < 0.05 Group A vs Group B

**Supplementary Table 4.**

**Glucagon units (any type) by country. Totals and mean ± SD. Retail and hospital sales are considered together,**

| **_Country_** | **_year_** | | | | | | | | _persons with diabetes_ |
| --- | --- | --- | --- | --- | --- | --- | --- | --- | --- |
|  | **_2014_** | **_2015_** | **_2016_** | **_2017_** | **_2018_** | **_2019_** | **_2020_** | **_2021_** |  |
| _ARGENTINA_ | _3,828_ | _4,227_ | _4,417_ | _4,881_ | _4,866_ | _4,703_ | _4,002_ | _3,933_ | 1_,_800_,_000 |
| _AUSTRALIA_ | _100,198_ | _89,088_ | _103,202_ | _96,324_ | _97,433_ | _101,738_ | _109,749_ | _113,334_ | 1_,_490_,_000 |
| _CANADA_ | _124,403_ | _135,124_ | _125,874_ | _148,990_ | _135,811_ | _161,134_ | _154,100_ | _186,052_ | 3_,_000_,_000 |
| _CHINA *_ | _14,454_ | _961_ | _868_ | _1,117_ | _1,100_ | _2,073_ | _1,840_ | _2,114_ | 141_,_000_,_000 |
| _Europe_ | ^1342653^ | ^1405971^ | ^1495494^ | ^1461966^ | ^1531069^ | ^1723965^ | ^1645388^ | ^1759687^ | _61,800,000_ |
| _IRELAND_ | _25,015_ | _27,113_ | _32,004_ | _29,400_ | _30,295_ | _35,876_ | _31,679_ | _32,898_ | 140_,_000 |
| _JAPAN_ | _1,632,908_ | _1,628,294_ | _1,563,591_ | _1,487,868_ | _1,418,351_ | _1,387,386_ | _1,227,445_ | _1,301,564_ | 11_,_000_,_000 |
| _N ZEALAND_ | _19,326_ | _19,355_ | _21,792_ | _19,717_ | _22,948_ | _23,021_ | _23,953_ | _24,839_ | 26_,_800 |
| _RUSSIA_ | _10,312_ | _3,611_ | _3,357_ | _7,173_ | _9,383_ | _10,428_ | _8,384_ | _17,920_ | 7_,_390_,_000 |
| _SAFRICA_ | _16,474_ | _17, ,197_ | _18,818_ | _16,757_ | _18,941_ | _19,252_ | _19,051_ | _21,147_ | 4_,_230_,_000 |
| _TURKEY_ | _71,641_ | _81,970_ | _86,213_ | _91,313_ | _99,398_ | _104,573_ | _94,937_ | _81,438_ | 9_,_020_,_000 |
| _UK_ | _213,188_ | _222,675_ | _239,591_ | _225,542_ | _226,544_ | _245,605_ | _230,894_ | _220,314_ | 3_,_990_,_000 |
| _US_ | _1,938,173_ | _1,886,496_ | _1,881,528_ | _1,874,486_ | _1,906,306_ | _1,953,805_ | _2,014,212_ | _2,252,508_ | 32_,_200_,_000 |
| *_TOTAL_*  *_mean_*  *_SD_* | *5,512,573*  *220,502*  *387,961* | *5,522,082*  *230,086*  *390,730* | *5,576,749*  *232,368*  *392,350* | *5,473,382*  *228,057*  *384,040* | *5,502,445*  *229,268*  *387,376* | *5,773,559*  *240,565*  *403,053* | *5,557,674*  *231,569*  *385,937* | *6,017,748 250,739*  *420,456* | *277,398,000 21,398,215 4,000,000* |

* data of China were not considered because of the unclear behaviour through different years

**Supplementary Table 5. Commercial sales of glucagon formulations (any type) by country. Retail and hospital sales are considered together,**

| **Country** | **Year** | | | | | | | | **Persons with diabetes** |
| --- | --- | --- | --- | --- | --- | --- | --- | --- | --- |
|  | **2014** | **2015** | **2016** | **2017** | **2018** | **2019** | **2020** | **2021** |  |
| ARGENTINA | 163_,_764 | 179_,_759 | 151_,_757 | 202_,_585 | 193_,_732 | 247_,_169 | 207_,_318 | 197_,_830 | 1_,_800_,_000 |
| AUSTRALIA | 3_,_159_,_397 | 2404149 | 2734299 | 2710036 | 2607907 | 2494229 | 2678197 | 2979970 | 1_,_490_,_000 |
| CANADA | 6_,_957_,_875 | 6_,_480_,_550 | 5_,_944_,_594 | 7_,_112_,_692 | 6_,_697_,_416 | 7_,_979_,_783 | 9_,_105_,_628 | 11_,_500_,_000 | 3_,_000_,_000 |
| CHINA * | 44_,_736 | 15,323 | 12,963 | 16,423 | 16,860 | 31,642 | 29,930 | 36,962 | 141_,_000_,_000 |
| EUROPE | 20_,_571_,_553 | 22_,_747_,_149 | 23_,_612_,_780 | 23_,_371_,_203 | 25_,_355_,_690 | 27_,_207_,_512 | 32_,_898_,_096 | 40_,_188_,_719 | 61_,_870_,_000 |
| IRELAND | 578_,_999 | 523_,_784 | 602_,_216 | 548_,_440 | 584_,_983 | 654_,_674 | 588_,_847 | 630_,_265 | 140_,_000 |
| JAPAN | 27_,_700_,_000 | 23_,_800_,_000 | 23_,_800_,_000 | 21_,_300_,_000 | 19_,_600_,_000 | 19_,_000_,_000 | 17_,_500_,_000 | 18_,_100_,_000 | 11_,_000_,_000 |
| N ZEALAND | 512_,_370 | 435_,_464 | 487_,_326 | 448_,_215 | 509_,_137 | 485_,_984 | 499_,_303 | 562_,_009 | 26_,_800 |
| RUSSIA | 151_,_576 | 32_,_676 | 27_,_376 | 65_,_173 | 78_,_327 | 85_,_481 | 62_,_440 | 130_,_873 | 7_,_390_,_000 |
| S AFRICA | 385_,_746 | 342_,_726 | 353_,_427 | 378_,_233 | 434_,_959 | 414_,_036 | 386_,_131 | 520_,_776 | 4_,_230_,_000 |
| TURKEY | 521_,_565 | 478_,_239 | 588_,_940 | 693_,_463 | 702_,_397 | 846_,_527 | 737_,_533 | 638_,_100 | 9_,_020_,_000 |
| UK | 3_,_543_,_784 | 3_,_432_,_943 | 7_,_412_,_590 | 2_,_927_,_799 | 6_,_785_,_864 | 3_,_158_,_772 | 2_,_990_,_748 | 3_,_054_,_711 | 3_,_990_,_000 |
| US | 257_,_000_,_000 | 309_,_000_,_000 | 257_,_000_,_000 | 326_,_000_,_000 | 337_,_000_,_000 | 349_,_000_,_000 | 383_,_000_,_000 | 435_,_000_,_000 | 33_,_220_,_000 |
| *Total* | *326*_,_*435*_,_*350* | *369*_,_*872*_,_*762* | *322*_,_*728*_,_*268* | *385*_,_*774*_,_*262* | *400*_,_*567*_,_*272* | *411*_,_*605*_,_*809* | *450*_,_*684*_,_*171* | *503*_,_*540*_,_*215* | *277,398,000* |
| *Mean* | *25*_,_*110*_,_*412* | *28*_,_*451*_,_*751* | *24*_,_*825,251* | *29,674,943* | *30,812,867* | *31*_,_*661*_,_*985* | *34,668,013* | *38,733,863* | *21,398,215* |
| *SD* | *67,900,000* | *81,700,000* | *66,780,000* | *86,300,000* | *89,100,000* | *92,400,000* | *101,000,00* | *115,000,000* | *4,000,000* |

Sales are expressed in US Dollars, Totals, mean ± SD
